# Assessment of habitat change on bird diversity and bird–habitat network of a Coral Island, South China Sea

**DOI:** 10.1186/s12862-021-01865-y

**Published:** 2021-07-06

**Authors:** Yingcan Li, Zhiwen Chen, Chao Peng, Guangchuan Huang, Hongyu Niu, Hongmao Zhang

**Affiliations:** Institute of Evolution and Ecology, School of Life Sciences, Central China Normal University, Wuhan, 430079 Hubei China

**Keywords:** Habitat roles, Birds, Bird–habitat network, Tropical coral island, Conservation

## Abstract

**Background:**

Understanding how island ecosystems change across habitats is a major challenge in ecological conservation under the conditions of habitat degradation. According to a 2-year investigation on Dong Island of the Paracel Islands, South China Sea, we assessed the roles of different habitats at the species level and community level of birds using topological and network analysis.

**Results:**

In addition to the thousands of *Sula sula* (a large-sized arboreal seabird) inhabiting the forests, there were 56 other bird species were recorded, representing 23 families and 12 orders, ranging in habitats of wetlands, forests, shrublands, grasslands, and/or beaches. The bird–habitat network had high nestedness, and bird species showed obvious clustering distribution. Integrated topological and network analysis showed that wetlands had a high contribution to species diversity and network structure, and it was a cluster center of migrant birds. Forests and grasslands were species hub and connector respectively, and forests were also the key habitat for residents. Beaches and shrublands were peripherals. The loss of wetlands and forests will result in a sharp reduction of species richness, and even make the *S. sula*, and most of the resident birds, become locally extinct.

**Conclusions:**

These results suggest that the wetland and forest habitats on the focal island are key important for migrant birds and resident birds respectively, and therefore much more attention should be paid to conservation of the focal island ecosystems.

**Supplementary Information:**

The online version contains supplementary material available at 10.1186/s12862-021-01865-y.

## Background

Oceanic islands are vital to the maintenance of biodiversity of the oceanic ecosystem because they serve as transfer stations for migrant birds and marine mammals, and have a great contribution to freshwater protection and local climate regulation [5, 10, 29, 55, 56]. However, oceanic island ecosystems have unique vulnerabilities due to their special geographical location, isolation, limited area, and other inherent factors [18]. In recent decades, the oceanic island ecosystems have been undergoing degradation due to serious anthropogenic disturbance, alien species invasion, and global climate changes [10, 24, 30, 42, 52]. Therefore, island conservation and restoration have become a worldwide concern [54, 56].

Under the influence of natural and anthropogenic factors, island ecosystems are experiencing the shrinkage or loss of habitats, the reduction or extinction of species in these corresponding habitats, and further the degradation of ecosystem function [14, 54, 56]. Oceanic islands generally represent global biodiversity hotspots harboring a high number of endemic and rare species prone to extinction [13]. Endemic species on islands often have a narrow distribution range and few available habitat types, and therefore are incapable of adapting to anthropogenic habitats under the conditions of land-use changes [54]. Thus, in order to protect these diverse and fragile island ecosystems, impacts of habitat loss on animal-habitat network structure need to be better understood.

In ecological networks, responses to nodes loss and how network properties affect extinction patterns have been widely concerned in recent years [8, 22, 23, 41]. Complex networks are generally considered error resistant, however, losing a few critical nodes might make these networks extremely to be vulnerable to attack [3]. From this point of view, by combining topology and network analysis, we can predict and evaluate robustness and structure of an island ecological network, and define the important habitat nodes as the major interactors that have many links to species. Their disruption may break relationships among sets of species, and therefore destroy ecosystem integrity and function.

Birds play a crucial role in marine island ecosystems, such as pollination [36, 53], seed dispersal [17, 20], and soil formation [1, 59]. Therefore, birds are often used as indicators in island ecosystems [26]. As the unique semi-closed environments, islands affect bird speciation and diversity according to their area, primary productivity, average annual temperature, distance to the mainland, and geological age [19, 35, 37, 56, 57]. The differences in habitat with respect to geographical location, vegetation type and resource are considered to affect the number and abundance of bird species [31, 44]. Wetlands on an island provide food, shelter, and stopover sites that allow birds to make migratory journeys [25], and the area of forest cover on an island is beneficial for the persistence of local bird species [49]. Therefore, bird–habitat network is an important indicator of the ecological function and health of the marine island ecosystems.

China’s Paracel Islands are located in the midwest part of the South China Sea, consisting of 32 islands, reefs, sandbanks, and shoals. These islands are rich in natural resources and inhabited by many species of birds, and therefore have immeasurable ecological and economic value [15, 16]. Although a part of the islands has been under the administration of nature reserves, the Paracel Islands are undergoing habitat degradation caused by invasive species and anthropogenic disturbance in recent years [38]. However, it is difficult to make decisions for ecological-based restoration and conservation to these islands due to the effects of habitat change on animal diversity and animal-habitat interactions are not fully known.

Dong Island is a typical island with continuous natural vegetation and mild disturbance of the Paracel Islands. From the center to the edge of Dong Island, the landcovers of forests, wetlands, shrublands, grasslands, and beaches are distributed sequentially but distichously between every two of them [46]. In the previous studies in 1974 and 2005, 43 and 55 bird species were recorded separately on the Paracel Islands. These birds are mainly migrant species (33 species, 76.74% in 1974, and 38 species, 69.09% in 2005), and usually utilize several specific habitat types (e.g., wetlands and forests) [33, 43]. Here, we used topological analysis to analyze the structure of bird–habitat network on Dong Island. We wanted to evaluate the role of nodes and habitats at both species and network levels. Probable effects of loss of each habitat type on bird species were assessed by removing habitats respectively and reanalyzing the bird–habitat networks. According to habitats’ conditions and historical data of bird composition on Dong Island, we hypothesized that (1) the bird community on Dong Island showed a clustering distribution, in that different migrant types had different clustering habitats; and (2) wetlands were of key importance for migrant birds, whereas forests were crucially important for resident birds, and these two habitats were key factors for the network structure. These results would be helpful to understand the ecological function and dynamic of island ecosystems, and to guide the planning of island ecosystems’ restoration and conservation.

## Results

### Bird diversity and bird–habitat network structure

**Fig. 1:**
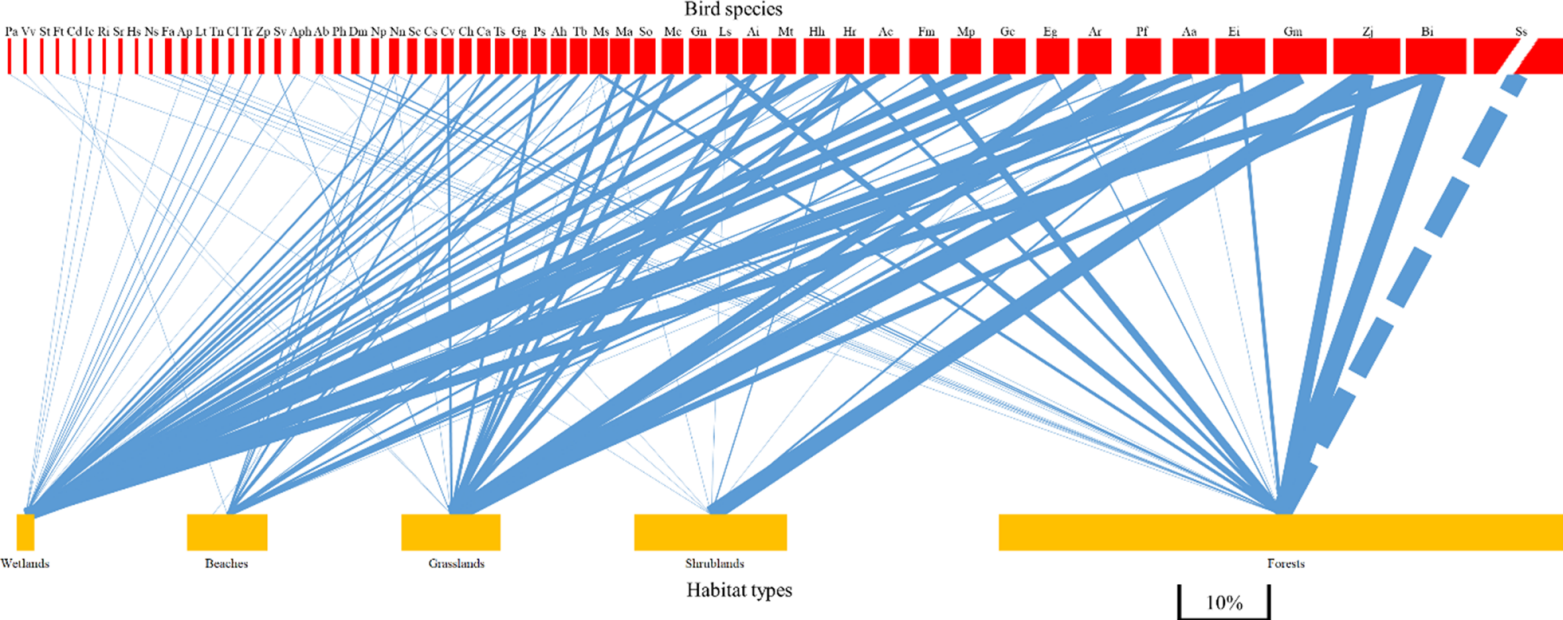
Bipartite representation of the bird–habitat network. The red column represents bird species and the yellow column represents habitats. The width of the columns indicates the proportion of birds or habitats in the total; the lines in the middle represent the relationship between birds and habitats; the width of the lines means relationship strength. Because of the large number of *Sula sula*, we choose dotted lines to represent it and its relationship. Bird species are Pa, *Phoenicurus auroreus*; Vv, *Vanellus vanellus*; St, *Saxicola torquate*; Ft, *Falco tinnunculus*; Cd, *Cecropis daurica*; Ic, *Ixobrychus cinnamomeus*; Ri, *Rallus indicus*; Sr, *Scolopax rusticola*; Hs, *Hierococcyx sparverioides*; Ns, *Ninox scutulata*; Fa, *Fregata ariel*; Ap, *Ardea purpurea*; Lt, *Lanius tigrinus*; Tn, *Tringa nebularia*; Cl, *Charadrius leschenaultia*; Tr, *Tachybaptus ruficollis*; Zp, *Zapornia pusilla*; Sv, *Sturnus vulgaris*; Aph, *Amanurornis phoenicurus*; Ab, *Ardeola bacchus*; Ph, *Pandion haliaetus*; Dm, *Dicrurus macrocercus*; Np, *Numenius phaeopus*; Nn, *Nycticorax nycticorax*; Sc, *Streptopelia chinenesis*; Cs, *Calidris subminuta*; Cv, *Charadrius veredus*; Ch, *Charadrius hiaticula*; Ca, *Charadrius alexandrinus*; Ts, *Tringa stagnatilis*; Gg, *Gallinago gallinago*; Ps, *Pluvialis squatarola*; Ah, *Actitis hypoleucos*; Tb, *Tringa brevipes*; Ms, *Monticola solitarius*; Ma, *Motacilla alba*; So, *Stretopelia orientalis*; Mc, *Motacilla cinerea*; Gn, *Gelochelidon nilotica*; Ls, *Lanius schach*; Ai, *Arenaria interpres*; Mt, *Motacilla tschutschensis*; Hh, *Himantopus Himantopus*; Hr, *Hirundo rustica*; Ac, *Ardea cinerea*; Fm, *Fregata mintor*; Mp, *Mareca Penelope*; Gc, *Gallinula chloropus*; Eg, *Egretta garzetta*; Ar, *Anthus richardi*; Pf, *Pluvialis fulva*; Aa, *Ardea alba*; Ei, *Egretta intermedia*; Gm, *Glareola maldivarum*; Zj, *Zosterops japonicus*; Bi, *Bubulcus ibis*; and Ss, *Sula sula*. Habitat types are W, wetlands; B, beaches; G, grasslands; S, shrublands; and F, forests

During the two experimental years, thousands of *S. sula* and a total of 1513 individuals of other species were recorded, belonging to 57 species, 23 families, and 12 orders (Additional file 1: Table S1). Among them, 20 species were resident birds (35.1%) and 37 species were migrant birds (64.9%). There were 103 links between birds and habitats, including 36 species in the wetlands, 22 species in the forests, 19 species in the grasslands, 17 species in the beaches, and nine species in the shrublands. Correlation analysis showed that species number has no significant correlation with habitat size (*P* = 0.88). The bird–habitat network exhibited low modularity (M (Modularity) = 0.33), high nestedness (NODF (Nestedness metric based on the Overlap and Decreasing Fill) = 42.79), and moderate connectance (C (Connectance) = 0.36) (Fig. 1).

Birds’ preference for habitats varied greatly on Dong Island, for instance, 63.2% of bird species were observed in the wetlands, while only 15.8% of bird species were observed in the shrublands (Table 1). Species number was highest in the wetlands and then decreased in the order of forests, grasslands, beaches, and shrublands. The Shannon–Wiener diversity index was highest in the wetlands, followed by the beaches, grasslands, shrublands, and forests. A greater proportion of migrant birds occurred in the wetlands and beaches than in the other habitats, while there were more resident birds in the forests than in other habitats. Pielou uniformity index was lower in the forests and shrublands than in the other habitats (Table 1).

**Table 1:** Bird species diversity and the basic parameters of bird–habitat network in each habitat on Dong Island, South China Sea

	Wetlands	Forests	Beaches	Grasslands	Shrublands
Bird species (%)	36 (63.16)	22 (38.60)	17 (29.82)	19 (33.33)	9 (15.79)
Resident birds (%)	12 (60)	13 (65)	5 (25)	13 (65)	5 (25)
Migrant birds (%)	24 (64.86)	9 (24.32)	12 (32.43)	6 (16.22)	4 (10.81)
Shannon–Wiener diversity index	2.86	0.41	2.43	2.27	0.58
Pielou uniformity index	0.80	0.13	0.86	0.77	0.26
Habitat strength	22.78	12.87	6.39	12.27	2.68
Interaction asymmetry	0.61	0.54	0.32	0.59	0.19
Nested rank	0	0.25	0.75	0.5	1
Specificity index	0.25	0.92	0.32	0.37	0.88

For species-level network parameters in the wetlands, habitat strength and interaction asymmetry were the highest, and nested rank and specificity index were the lowest (Table 1), suggesting that the wetlands were a key node for bird species and networks, and had the highest contribution to both the bird community and network structure. In the forests and grasslands, habitat strength and interaction asymmetry were relatively high, while nested rank were medium or low, suggesting that these habitats had a relatively high contribution to bird species and network structure. In the beaches and shrublands, the low level of habitat strength and interaction asymmetry, and a high nested rank indicated a relatively low status in the network and limited species contribution. Shrublands and forests had the highest specificity index due to the few numbers of bird species and the largest number of *S. sula* individuals respectively.

**Fig. 2:**
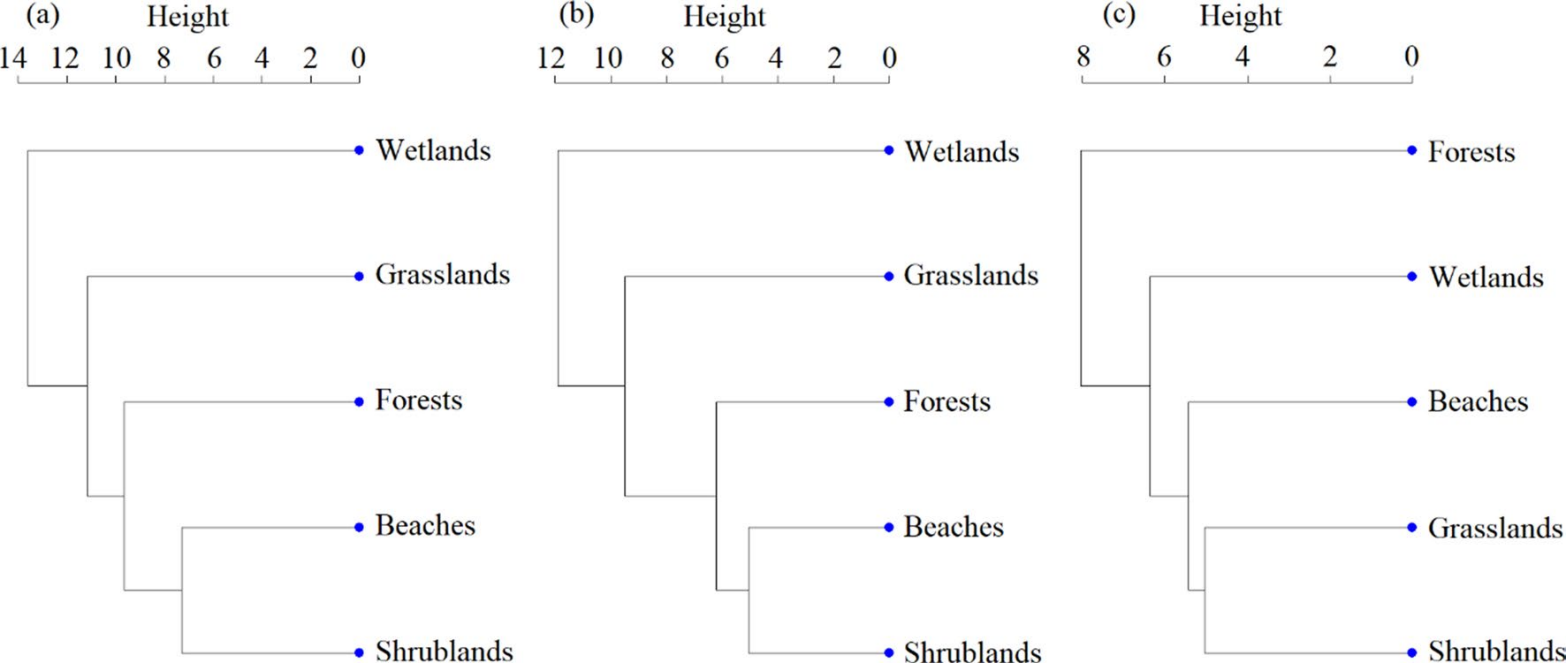
The similarity tree of bird species in different habitats on Dong Island. **a** The similarity tree of total bird species; **b** The similarity tree of migrant bird species; **c** The similarity tree of resident bird species. The height in the similarity tree represents the relative distance and the distinction between habitat types

The similarity trees of resident birds, migrant birds and in total showed insignificant difference among habitats (Fig. 2). The similarity sequences of total bird species and migrant bird species were the same, for example, the shrublands had the closest similarity with the beach, and the most dissimilar with the wetlands. While in the similarity tree of resident bird species, the closest and furthest similarity from the shrubland were those of the grasslands and forests, respectively.

In the topological approach, the sequence removing the smallest habitat shows the lowest robustness (Additional file 1: Fig. S1). First, the bird–habitat network was less robust to the sequences removing the most connected and the smallest habitat as compared to the random sequence. Second, the bird–habitat network was more robust against the sequences removing the least connected and the biggest habitat than the random sequence.

### Effects of presumed habitat loss

**Fig. 3:**
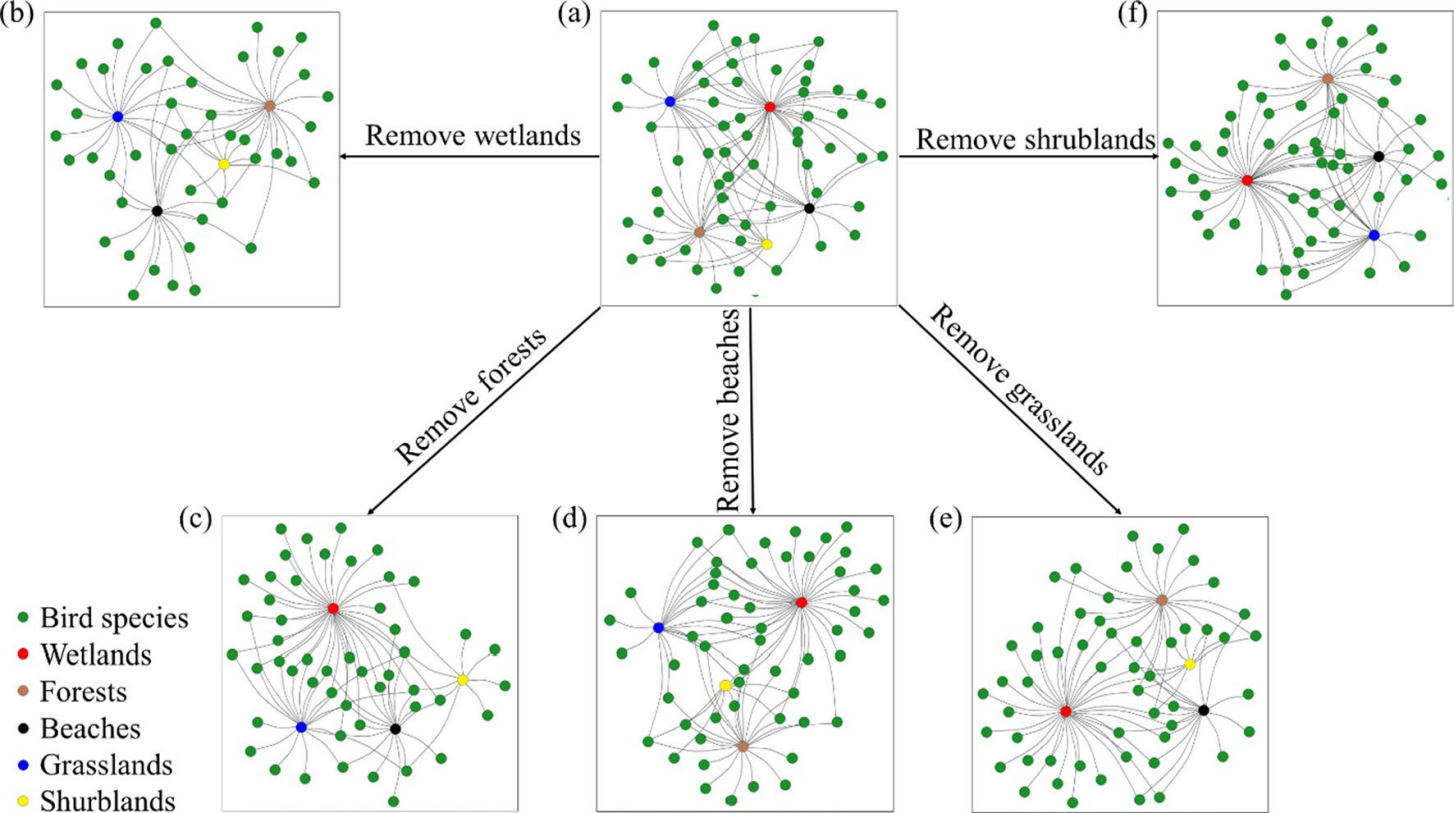
Bipartite representation of the bird–habitat network on Dong Island, South China Sea. **a** The structure of the network including all birds and habitats. **b** The structure of the network when wetlands were removed. **c** The structure of the network when forests were removed. **d** The structure of the network when beaches were removed. **e** The structure of the network when grasslands were removed. **f** The structure of the network when shrublands were removed. The nodes represent habitat types and bird species, the lines represent the bird species distribution in the habitats

**Fig. 4:**
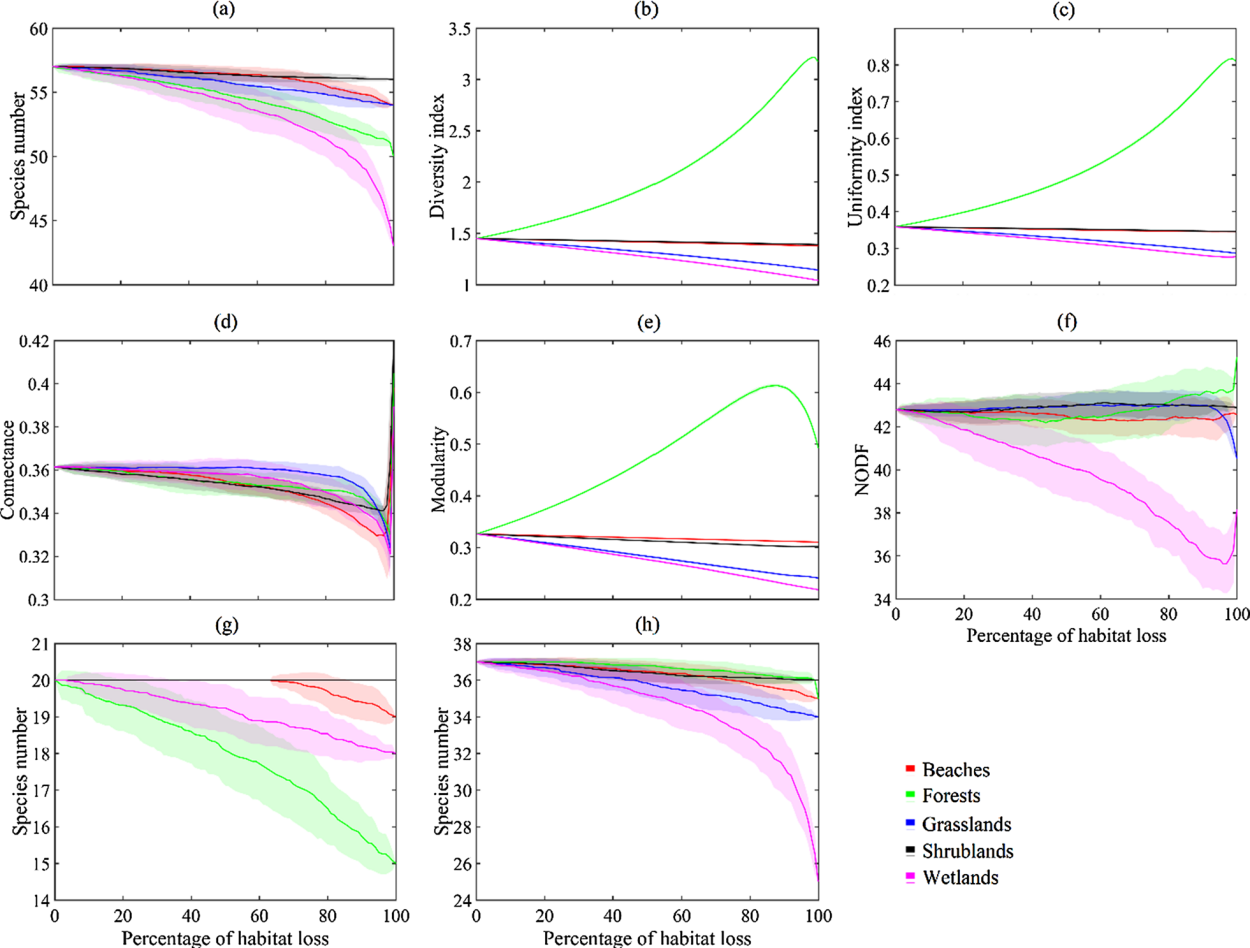
Effects of presumed habitat loss on the bird diversity and bird–habitat network structure on Dong Island, South China Sea. **a** species number; **b** Shannon–Wiener diversity index; **c** Pielou uniformity index; **d** Connectance of the bird–habitat network; **e** Modularity of the bird–habitat network; **f** NODF of the bird–habitat network; **g** Species number of resident birds; **h** Species number of migrant birds

We used the topological method to simulate habitat loss in the network (Fig. 3). Bird species on Dong Island were greatly reduced with the increase of habitat loss of the wetlands and forests (Fig. 4a, Additional file 1: Fig. S2a), indicating many species only utilized these habitats. Shannon–Wiener diversity of birds increased greatly with the increasing percentage of habitat loss of the forests, while the index decreased with the loss of the wetlands, grasslands, or beaches when *S. sula* was excluded (Fig. 4b, Additional file 1: Fig. S2b). Pielou uniformity index increased sharply with increased loss of the forests, while the index decreased with the loss of the wetlands or grasslands when *S. sula* was excluded (Fig. 4c, Additional file 1: Fig. S2c). Simulation of species parameters indicated that the birds mainly utilized the habitats of the wetlands, grasslands, and beaches, while the shrublands and beaches had a relatively low population of birds.

For network structure, removal of each habitat had a similar effect on connectance (Fig. 4d, Additional file 1: Fig. S2d), while differed on modularity and nesting. Removal of the forests caused an increase of modularity of the bird–habitat network. In contrast, when *S. sula* were excluded, removal of the grasslands caused a great reduction of modularity, and removal of the beaches or forests caused a small increase and decrease, respectively, and removal of the wetlands and shrublands had little effects on the modularity of the network (Fig. 4e, Additional file 1: Fig. S2e). Removal of the forests caused the network to be more nested, while removal of the wetlands caused a great reduction of nestedness. Meanwhile, removal of the beaches or grasslands caused a little reduction of nestedness, whereas, removal of the shrublands caused a little effect on nestedness (Fig. 4f, Additional file 1: Fig. S2f).

For migrant birds, removal of the wetlands caused a loss of 12 species (32.4%), and then decreased with the order of the grasslands (three species, 8.1%), beaches (two species, 5.4%), forests (two species, 5.4%) and shrublands (one species, 2.7%) (Fig. 4g, Additional file 1: Fig. S2g). For resident species, removal of the forests caused a loss of five species (25.0%), removal of the wetlands (two species, 10.0%) or beaches (one species, 5.0%) caused light effects, while removal of the grasslands or shrublands had no effects on resident bird species (Fig. 4h, Additional file 1: Fig. S2h).

## Discussions

Our results suggest that bird species showed clustering distribution and were not related to habitat area, and different habitats played different roles in bird–habitat network structure, supporting our previous hypothesis 1: the bird community on Dong Island showed a clustering distribution, in that different migrant types had different clustering habitats. Among all of the habitats, removal of grasslands reduced modularity of the network to the greatest extent. Nestedness of the network decreased greatly when the wetlands were removed, while it increased by the loss of the forests. Migrant birds primarily utilized the wetlands, and resident birds predominantly inhabited the forests. As hypothesis 2 predicted, both the wetlands and forests provide a great contribution to the structure and biodiversity of the bird–habitat network on Dong Island, and therefore they should receive more attention in conservation.

Compared to the other islands of the study area, Dong Island has a relatively high species richness and diversity of birds, showing a clustered distribution unrelated to habitats’ area. Many species only utilized one or two habitats. For example, *Egretta intermedia* were common in the wetlands, *S. sula*, *Bubulcus ibis* mainly utilized the forests, *Zosterops japonicus* were mainly distributed in the shrublands, *Glareola maldivarum* were easily found in the grasslands, and *Ardea alba* were common to see on the beaches. On Dong Island, Pan [43] reports there are 42 species of birds (19 families, 10 orders, 12 resident birds and 30 migrant birds). Cao et al. [15] report there are more than 35,500 pairs of *S. sula* inhabiting the forests on Dong Island. Compared to these studies, our results suggest that species richness and diversity of birds have a slight increase, and the structure of the bird community changed accordingly. With the reduction of anthropogenic disturbance and restoration of vegetation (e.g., planting trees and grass), habitats on Dong Island are being made more suitable for bird breeding [15]. Compared to the previous studies [43], species of Coraciiformes, Ciconiiformes, Accipitridae, and Cettiidae were not recorded, and species of Aniseriformes, Podicipediformes, Columbiformes, Cuculiformes, Strigiformes, Pandionidae, Dicruridae, Laniidae, Sturnidae, and Muscicapidae were firstly recorded this time. The smallest wetland contained the highest species richness, while the shrublands, the second-largest habitat, had the least number of species, and the forests contained almost all of *S. sula* and other resident birds. In contrast to the species-area relationship in that species richness is positively correlated to habitat area size, the non-uniform distribution of birds on Dong Island might be caused by the unique food availability, shelter, nest sites, and species interactions in each habitat [28, 58].

The similarity between habitats was relatively low, and the similarity tree of total bird species was dominated by migrant birds (Fig. 2). There are obvious differences in similarity tree between migrant and resident bird community. The different patterns represent each resident type has its own habitat preference. The migrant bird community is mainly consisted of wading birds (such as shorebirds and egrets), and their typical habitat is wetlands such as tidal flats and swamps. The resident bird community is mainly consisted of songbirds, wading birds and raptors, they prefer habitats that provide safe shelter or food resources such as insects or plant seeds.

Loss of habitat on islands usually leads to threatened and extinction of endemic species, as well as simplification and fragility of ecological networks [14, 42]. Our results showed that different types of habitat played various roles in species and network levels, and effects of habitat loss on species and network structure had been predicted. Loss of wetlands had the greatest impact on diversity index, species number, and uniformity index, and greatly reduced nestedness in the bird–habitat network, which would cause serious loss of biodiversity and simplification of the ecological network. This loss of biodiversity occurs because, on such an oceanic coral island, the wetland habitat serves as a community hub, and typically supports a variety of biota, many of which are wetland specific species, by providing fresh water, improving water quality and providing additional habitat structure with its plants [6, 60]. Loss of the grasslands had a moderate impact on diversity index, species number, and greatly reduced uniformity index and modularity of the network, which would cause moderate loss of biodiversity but serious network instability. Considering *S. sula*, loss of the forests greatly changed the diversity, uniformity, and modularity. In contrast, it had a serious impact on species number and slight incremental addition of nestedness when *S. sula* was excluded. Therefore, grasslands indicate that it is a connector, while the forests present a species hub. As for the shrublands or beaches, their removal had little impact on both diversity and network structure. Such peripherals contributed little to the entire ecosystem and had little impact on network structure or species diversity.

China’s Paracel Islands are located in the East Asian-Australasian Flyway (EAAF), one of the four globally recognized flyways for migratory birds [12]. Birds in the EAAF are facing complex and formidable threats, and habitat loss is a serious problem [47]. On Dong Island, the wetlands contain the maximum and most unique migrant species, and its loss would seriously affect the migrant richness and ecological function. Forests are not only the key habitat for the resident birds of Dong Island but also provide the only breeding ground and habitat for the largest breeding population of the *S. sula* in the Western Pacific [15]. Thus, the forests on Dong Island is crucially important and, therefore, is integral to conservation.

## Conclusions

Dong Island had a relatively high richness of birds and habitats, and the birds tended to cluster their distribution and had high diversity in some narrow habitats (e.g., wetlands and grasslands). The wetlands were a community hub of the bird–habitat network, the forests and grasslands were species hubs and connectors respectively, and the beaches and shrublands were peripherals. The wetlands were crucial for migrant species, while the forests were essential for resident species, especially for *S. sula*, the largest breeding population in the Western Pacific. Our study on bird–habitat network provides a possibility to examine community-driven, robust patterns of habitat loss in tropical coral island ecosystems, and may be applicable to other types of ecological networks. The network responses of simulated habitat loss may provide novel insights into its stability from a structural perspective, which helps to explain the role of habitats in the island ecosystems, and highlights that integration of topology and network analysis is practical to assess conservation objectives.

## Methods

### Study site

We conducted this study on Dong Island of the Paracel Islands, China. The area is 171 ha, making it the second-largest island in the Paracel Islands. The elevation is about 3–6 m, and the highest point is 6.7 m. The shoreline is 6.12 km in length, and the distance between Dong Island and the mainland of China is about 337 km [48]. The Paracel Islands have a tropical monsoon climate, with a mean annual temperature of 26 ℃, mean annual precipitation of 1500 mm, mean annual sunlight of 2900 h, and mean annual relative humidity of 81%. The dry season is from December to May, and the rainy season is from June to November, accounting for 87% of the annual rainfall [39]. The island is under state protection and maintains the best natural vegetation in the Paracel Islands, but the habitats are still undergoing degradation due to the invasion of alien species (e.g., *Chromolaena odorata*, *Sphagneticola lobata*, *Rattus flavipectus*), garbage accumulation from ocean currents, global climate change, and other threats, which puts high pressure on the island ecosystem [15, 46].

### Habitat type

There are five main habitat types on Dong Island. The largest vegetation type is broad-leaved evergreen forests, which have an area of 99 ha and greatly distributed in the core of the island. The common trees are *Ceodes grandis* and *Guettarda speciose*, with 4–10 m height, 70–80% canopy cover. There are very few undergrowth vegetations under forests. The second largest vegetation type, shrublands, is about 20.2 ha, mainly distributed along the coast of the island. The shrubs are 1–2.5 m in height and 55–75% in coverage, and dominated by *Scaevola sericea* and *Messerschmidia argentea*. The grasslands are about 19.0 ha in area size, distributed on the beaches of the high tide line of the coastal front and in the open lands of the island, dominated by herbaceous species *Ipomoea pescaprae*, liana species *Tridax procumbens*, *Portulaca oleracea*, and *Zoysia matrella*. The beaches are about 11.2 ha, distributed on the edge of the island, and support growth of a few salt-tolerant plants. Whereas the wetland is a naturally brackish-water lake and the shallow-water areas around it, about 2.2 ha in size, located at the south of the island, and dominated by two species of limnophyte, *Paspalum longifolium* and *Sesuvium portulacastrum*.

### Bird survey

Four line-transects were sampled for bird survey on the whole island. Each transect was 800–1000 m in length, passed through all types of habitats, and the sampled areas covered more than 80% of the island. In each transect, five sites with an interval of 150–200 m were selected for point counts, and the location of each site was recorded by a GPS of mobile phone (Huawei Mate30 Pro + , HUAWEI TECHNOLOGIES CO., LTD., China).

Each transect was repeatedly investigated at least 10 times in each season (dry and rainy season) from May 2018 to December 2019. Field observations were conducted at 0600–0900 h and 1600–1900 h on each survey day. For each survey, two people walked along each transect at a speed of 2–3 km/h to observe birds using binoculars (Kowa, 10 × 42, made in Japan) and telephoto cameras (Nikon P900s, made in Japan). A total of 20–30 min was spent for point count at each site. For each observation, bird species, the number of individuals, and their habitats were recorded. Birds were identified and classified according to A Field Guide to the Birds of China [40], and A Checklist on the Classification and Distribution of the Birds of China (3rd edition) [61].

### Bird–habitat networks

We used the following parameters to characterize bird diversity and bird–habitat network through *bipartite* package [21] in R 3.4.2 [45]:

1. Richness, the number of bird species;
2. Shannon–Wiener diversity index and Pielou uniformity index, parameters to measure abundance and heterogeneity, were mainly used in the diversity characteristics of birds [32],
3. Connectance (C), the proportion of realized interactions out of those possible in the network [22],
4. Nestedness (NODF, Nestedness metric based on the Overlap and Decreasing Fill), which describes a pattern in which birds in habitats with few bird species were subsets of those in habitats with many bird species [4],
5. Modularity (M), refers to subsets (module) of closely interacting species, which has relatively little or no interaction with other subsets [27],
6. Habitat strength, which refers to the sum of the action intensity of a particular habitat on species [7],
7. Nested rank, which refers to the level of a network nested matrix, with the lower the value, the higher the universality, and vice versa [2],
8. Specificity index, also known as specialization, is used to measure the degree of specialization, the more species interact with it, the lower the specificity index [11],
9. Interaction asymmetry, refers to the species asymmetry of interaction; positive value represents that a particular habitat has greater dependence or influence on species, and a negative value represents that a particular habitat is dependent or influenced by species [7, 11].

Forms of computation of these parameters were presented as supplement methods (Additional file 1: additional methods).

We used ANOVA and correlation analysis for data processing, and used hierarchical clustering analysis to construct similarity trees. All data analyses were performed in R 3.4.2 [45].

### Simulation of habitat loss

A weighted bird–habitat network was described by a type of adjacency matrix called flux matrix A [9]. Each non-zero element A_[*i, j*]_ represents the number of bird *j* visiting habitat *i*. Column *j* represents the chosen habitats of bird species *i*. For simplicity, we assumed that birds cannot switch from one type of habitat to another after habitat loss [51]. In the binary extinction scenario, a bird species *j* goes extinct when the normalized column sum of the *j* column, that is, species *j* goes extinct when there is a lack of available habitat [22, 23, 50]. We performed simulations in which a certain percentage of habitat was randomly removed at each step and the number of secondary extinctions was recorded. The procedure was repeated until all the nodes were lost.

In the simulation of the impact of habitat loss on bird–habitat network structure, where secondary extinctions occur when a species was left without any exploitable resources [34]. The extinction threshold was not considered because birds on oceanic islands have a strong dispersal capacity [8].

Because there are more than 35,500 pairs of *S. sula* inhabit the forest on Dong Island [15], which concealed the effects of habitat loss when using the Shannon–Wiener diversity, Pielou uniformity, and modularity analyses, we repeated these analyses by excluding *S. sula*.

## Supplementary Information

**Additional file 1: Table S1.** Bird species on Dong Island, South China Sea. **Fig. S1.** The robustness to habitat loss in the topological approach. On the y-axis is robustness measured as R50. Each box plot contains the R50 values from the 50 repeated simulations. Lower case letters above the box plots denote significantly different sequences (different letters) in the ANOVA at the 0.05 level. **Fig. S2.** Effects of presumed habitat loss on the bird diversity and bird–habitat network structure on Dong Island, South China Sea when *Sula sula *was excluded. (a), species number; (b), Shannon-Wiener diversity index; (c), Pielou uniformity index; (d), Connectance of the bird–habitat network; (e), Modularity of the bird–habitat network; (f), NODF of the bird–habitat network; (g), Species number of resident birds; (h), Species number of migrant birds.

## Data Availability

Data sources for bird species and bird–habitat relationship for our study can be found in Additional file 1: Table S1.

## Abbreviations

C: Connectance
NODF: Nestedness metric based on the overlap and decreasing fill
M: Modularity
