# Supplementary material for: Assessment of habitat change on bird diversity and bird–habitat network of a Coral Island, South China Sea
Source: BMC Ecol Evol. 2021 Jul 6;21:137. doi: 10.1186/s12862-021-01865-y (PMC8259361; doi:10.1186/s12862-021-01865-y)
Supplement: Supplementary file 1 — Additional file 1: Table S1. Bird species on Dong Island, South China Sea. Fig. S1. The robustness to habitat loss in the topological approach. On the y-axis is robustness measured as R50. Each box plot contains the R50 values from the 50 repeated simulations. Lower case letters above the box plots denote significantly different sequences (different letters) in the ANOVA at the 0.05 level. Fig. S2. Effects of presumed habitat loss on the bird diversity and bird–habitat network structure on Dong Island, South China Sea when Sula sula was excluded. (a), species number; (b), Shannon-Wiener diversity index; (c), Pielou uniformity index; (d), Connectance of the bird–habitat network; (e), Modularity of the bird–habitat network; (f), NODF of the bird–habitat network; (g), Species number of resident birds; (h), Species number of migrant birds. [file 12862_2021_1865_MOESM1_ESM.docx]

Table S1 Bird species on Dong Island, South China Sea.

| Order | Family | Species | Relative abundance | Residence type | Fauna | Habitat | IUCN |
| --- | --- | --- | --- | --- | --- | --- | --- |
| Aniseriformes | Anatidae | Eurasian wigeon, *Mareca Penelope* | ++ | W | P | W | LC |
| Podicipediformes | Podicipedidae | little grebe, *Tachybaptus ruficollis* | + | R | W | W | LC |
| Columbiformes | Columbidae | oriental turtle dove, *Stretopelia orientalis* | ++ | R | W | GW | LC |
|  |  | spotted dove, *Streptopelia chinenesis* | + | R | O | BG | LC |
| Cuculiformes | Cuculidae | large hawk cuckoo, *Hierococcyx sparverioides* | + | R | O | F | LC |
| Gruiformes | Rallidae | brown-cheeked rail, *Rallus indicus* | + | V | P | W | LC |
|  |  | Baillon's crake, *Zapornia pusilla* | + | W | W | W | LC |
|  |  | white-breasted waterhen, *Amanurornis phoenicurus* | + | R | O | FGSW | LC |
|  |  | common moorhen, *Gallinula chloropus* | ++ | W | W | GW | LC |
| Charadriiformes | Recurvirostridae | black-winged stilt, *Himantopus himantopus* | ++ | P | W | BW | LC |
|  | Charadriidae | northern lapwing, *Vanellus vanellus* | + | W | W | G | NT |
|  |  | little ringed plover, *Charadrius dubius* | + | W | W | W | LC |
|  |  | oriental plover, *Charadrius veredus* | + | V | P | BG | LC |
|  |  | Kentish plover, *Charadrius alexandrinus* | + | R | W | BW | LC |
|  |  | greater sand plover, *Charadrius leschenaultii* | + | W | P | BW | LC |
|  |  | pacific golden plover, *Pluvialis fulva* | ++ | W | P | BGW | LC |
|  |  | grey plover, *Pluvialis squatarola* | ++ | W | P | GW | LC |
|  | Scolopacidae | Eurasian woodcock, *Scolopax rusticola* | + | W | P | F | LC |
|  |  | common snipe, *Gallinago gallinago* | + | W | P | W | LC |
|  |  | Whimbrel, *Numenius phaeopus* | + | W | P | B | LC |
|  |  | marsh sandpiper, *Tringa stagnatilis* | + | W | P | W | LC |
|  |  | common greenshank, *Tringa nebularia* | + | W | P | W | LC |
|  |  | grey-tailed tattler, *Tringa brevipes* | ++ | W | P | BW | NT |
|  |  | common sandpiper, *Actitis hypoleucos* | ++ | W | P | BGW | LC |
|  |  | ruddy turnstone, *Arenaria interpres* | ++ | W | P | BGW | LC |
|  |  | long-toed stint, *Calidris subminuta* | + | W | P | W | LC |
|  | Glareolidae | oriental pratincole, *Glareola maldivarum* | +++ | P | W | BGW | LC |
|  | Laridae | gull-billed tern, *Gelochelidon nilotica* | ++ | P | W | W | LC |
| Suliformes | Suilidae | red-footed booby, *Sula sula* | ++++ | S | W | F | LC |
|  | Fregatidae | lesser frigatebird, *Fregata ariel* | + | S | W | F | LC |
|  |  | great frigatebird, *Fregata mintor* | ++ | S | W | BF | LC |
| Pelecaniformes | Ardeidae | cinnamon bittern, *Ixobrychus cinnamomeus* | + | R | W | W | LC |
|  |  | black-crowned night heron, *Nycticorax nycticorax* | + | R | W | BFGW | LC |
|  |  | Chinese pond heron, *Ardeola bacchus* | + | R | O | FSW | LC |
|  |  | cattle egret, *Bubulcus ibis* | +++ | R | O | BFGW | LC |
|  |  | grey heron, *Ardea cinerea* | ++ | W | W | BW | LC |
|  |  | purple heron, *Ardea purpurea* | + | W | W | FW | LC |
|  |  | great egret, *Ardea alba* | ++ | R | W | BFGW | LC |
|  |  | intermedia egret, *Egretta intermedia* | +++ | W | W | BFGSW | LC |
|  |  | little egret, *Egretta garzetta* | ++ | R | W | BFSW | LC |
| Accipitriformes | Pandionidae | osprey, *Pandion haliaetus* | + | R | W | F | LC |
| Strigiformes | Strigidae | brown boobook, *Ninox scutulata* | + | R | W | F | LC |
| Falconiformes | Falconidae | common kestrel, *Falco tinnunculus* | + | R | W | F | LC |
| Passeriformes | Dicruridae | black drongo, *Dicrurus macrocercus* | + | R | O | F | LC |
|  | Laniidae | tiger shrike, *Lanius tigrinus* | + | W | P | FS | LC |
|  |  | long-tailed shrike, *Lanius schach* | ++ | R | W | FS | LC |
|  | Hirundinidae | barn swallow, *Hirundo rustica* | ++ | W | W | BFGSW | LC |
|  |  | red-rumped swallow, *Cecropis daurica* | + | P | W | B | LC |
|  | Zosteropidae | Japanese white-eye, *Zosterops japonicus* | +++ | R | P | FS | LC |
|  | Sturnidae | common starling, *Sturnus vulgaris* | + | V | W | FG | LC |
|  | Muscicapidae | Daurian redstart, *Phoenicurus auroreus* | + | W | W | S | LC |
|  |  | Siberian stonechat, *Saxicola torquata* | + | W | W | G | LC |
|  |  | blue rock thrush, *Monticola solitarius* | ++ | W | W | BFGSW | LC |
|  | Motacillidae | Eastern yellow wagtail, *Motacilla tschutschensis* | ++ | W | W | GW | LC |
|  |  | grey wagtail, *Motacilla cinerea* | ++ | R | W | BGW | LC |
|  |  | white wagtail, *Motacilla alba* | ++ | R | W | BGW | LC |
|  |  | Richard's pipit, *Anthus richardi* | ++ | P | O | FG | LC |

Relative abundance: +, 1-10; ++, 11-100; +++, 101-1000; ++++, >1000. Residence type: R, resident; S, summer visitor; W, winter visitor; P, passage migrant; V, vagrant visitor. Fauna: P, Palearctic species; O, Oriental species; W, widespread species. Habitat: F, forests; S, shrublands; G, grasslands; B, beaches; W, wetlands. IUCN, International Union for Conservation of Nature: LC, least concern; NT, near threatened.


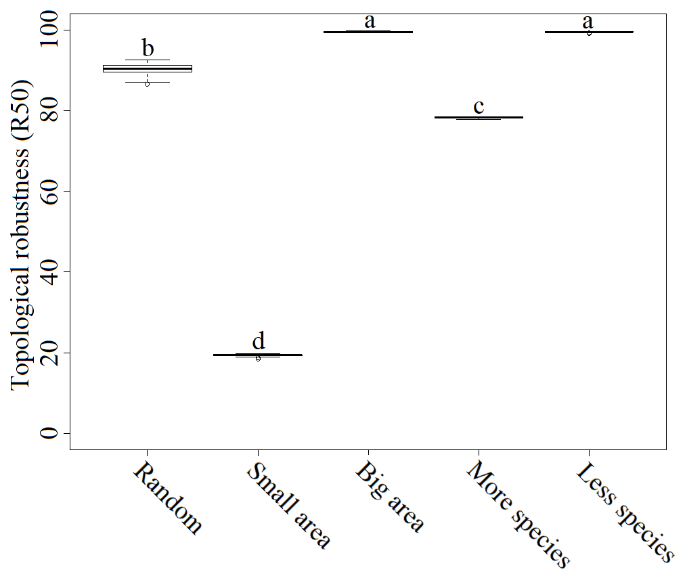


Fig. S1 The robustness to habitat loss in the topological approach. On the y-axis is robustness measured as R50. Each box plot contains the R50 values from the 50 repeated simulations. Lower case letters above the box plots denote significantly different sequences (different letters) in the ANOVA at the 0.05 level.


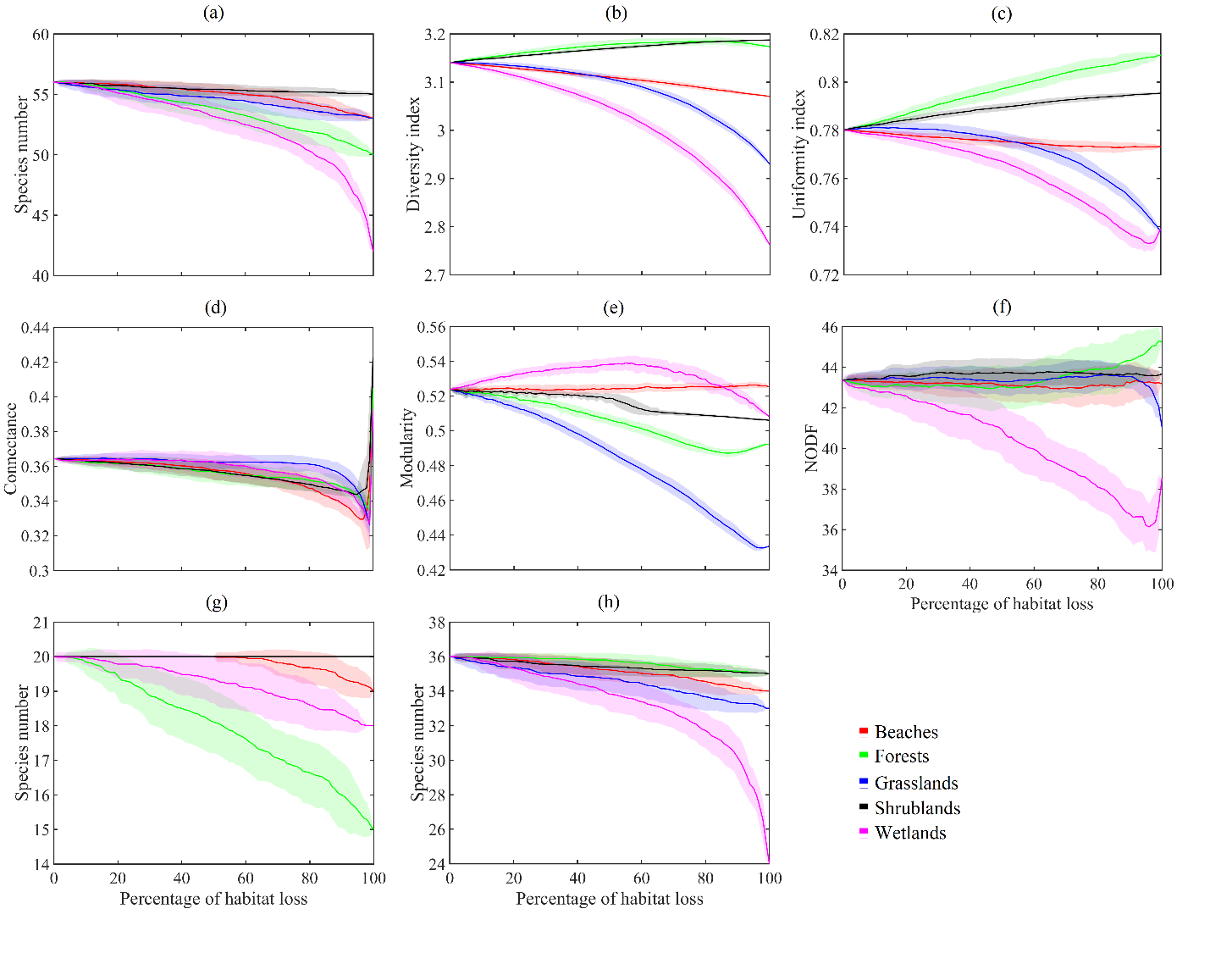


Fig. S2 Effects of presumed habitat loss on the bird diversity and bird–habitat network structure on Dong Island, South China Sea when *Sula sula* was excluded. (a), species number; (b), Shannon-Wiener diversity index; (c), Pielou uniformity index; (d), Connectance of the bird–habitat network; (e), Modularity of the bird–habitat network; (f), NODF of the bird–habitat network; (g), Species number of resident birds; (h), Species number of migrant birds.

Supplement methods

1. Richness is the species number of birds in the network;

2. Shannon-Wiener diversity index (*H’*): *H’*=-∑(*P_i_*)(ln*P_i_*). *P_i_* is the sum of individuals of species *i* divided by the total number of individuals in the network;

3. Pielou uniformity index (*J*): *J*=(-∑*P_i_*ln*P_i_*)/ln*S*. *P_i_* is the sum of individuals of species *i* divided by the total number of individuals in the network. *S* is the species number in the network;

4. Connectance (*C*): sum of links divided by the total number of possible links (=species number times habitat types);

5. Nestedness (NODF, Nestedness metric based on the Overlap and Decreasing Fill):

$$NODF=\frac{\sum N_{\mathrm{pa}\mathrm{ired}}}{\left[ \frac{n(n-1)}{2} \right]+\left[ \frac{m(m-1)}{2} \right]}$$

If DF_paired_ = 0, N_paired_ = 0; if DF_paired_ = 100, N_paired_ = PO.

Construct qualitative matrix according to network, 1 indicate that there is a link, 0 indicate that there is no link. *n* is the number of columns, and *m* is the number of rows. For any pair of rows *i* and *j*, if MT*_j_*< MT*_i_*, DF*_ij_* = 100, and if MT*_j_*≤ MT*_i_*, DF*_ij_* = 0. For any pair of columns k and l, if MT*_l_*< MT*_k_*, DF*_kl_* = 100, and if MT*_l_*≥ MT*_k_*, DF*_kl_* = 0.

For nestedness among rows, PO*_ij_* is the percentage of 1’s in a given row *j* that are in the same row as 1’s observed in row *i*. For nestedness among columns, paired overlap (PO*_kl_*) is the percentage of 1’s in a given column *l* that are in the same row as 1’s observed in column *k*.

6. Modularity (M)

$$M=\frac{1}{2m}\sum_{ij} {(A}_{ij}-K_{ij})\delta(c_{i},c_{j})$$

*m* = ∑*_i,j_A_ij_*; *A_ij_* is a weighted edge matrix; *K_ij_* is the expected weights for each link based on a null model; *c_i_* or *c_j_* is the module which species *i* or *j* assigned; if *c_i_* = *c_j_*, *ẟ*(*c_i_*, *c_j_*) = 1, and if *c_i_* ≠ *c_j_*, *ẟ*(*c_i_*, *c_j_*) = 0.

7. Habitat strength: the sum of the action intensity of a particular habitat on species;

8. Nested rank: the level of a network nested matrix. We set a ranking (1, 2, 3, …) for habitats, the most generalized habitat ranked 1, the next most generalized habitat ranked 2, and so on. We standardized the rank values by dividing them by the total number of habitat types;

9. Specificity index (*d’*):

$$d_{i}=\sum_{j=1}^{c} \left( p_{ij}^{'}ln\frac{p_{ij}^{'}}{q_{j}} \right)$$

*c* is the number of resources; *p’_ij_* is the ratio of the number of links divided by the sum of performances of species *i*; *q_j_* is the number of links of resource *j* divided by the total number of links in the network. And the specificity *d’* = (*d_i_* - *d_min_*)/ (*d_max_* - *d_min_*).

10. Interaction asymmetry (*A_i_*):

$$s_{ij}=\frac{f_{ij}}{\sum_{m=1}^{I} f_{mj}}$$

*s_ij_* is the strength of the effect of a species *i* in one group of the bipartite network on a species *j* in the second group; *f_ij_* is the frequency of interaction between pairs of species. *I* is the total number of species in the first group. The difference between the elements of two matrices *d_ij_* = *s_ij_* – *s_ji_*. And the interaction asymmetry is

$$A_{i}=\frac{\sum_{j=1}^{J} d_{ij}}{k_{i}}$$

Where *k_i_* is the number of links of species *i*.
